# Reducing the Immunogenic Potential of Wheat Flour: Silencing of Alpha Gliadin Genes in a U.S. Wheat Cultivar

**DOI:** 10.3389/fpls.2020.00020

**Published:** 2020-02-25

**Authors:** Susan B. Altenbach, Han-Chang Chang, Matthew H. Rowe, Xuechen B. Yu, Annamaria Simon-Buss, Bradford W. Seabourn, Peter H. Green, Armin Alaedini

**Affiliations:** ^1^Western Regional Research Center, United States Department of Agriculture-Agricultural Research Service, Albany, CA, United States; ^2^Takara Bio USA, Inc., Mountain View, CA, United States; ^3^Department of Medicine, Columbia University, New York, NY, United States; ^4^Institute of Human Nutrition, Columbia University, New York, NY, United States; ^5^Hamburg School of Food Science, Institute of Food Chemistry, University of Hamburg, Hamburg, Germany; ^6^Hard Winter Wheat Quality Laboratory, Center for Grain and Animal Health Research, United States Department of Agriculture-Agricultural Research Service, Manhattan, KS, United States; ^7^Celiac Disease Center, Columbia University, New York, NY, United States; ^8^Department of Medicine, New York Medical College, Valhalla, NY, United States

**Keywords:** alpha gliadins, celiac disease, gluten proteins, immunogenic potential, proteomics, wheat flour quality

## Abstract

The alpha gliadins are a group of more than 20 proteins with very similar sequences that comprise about 15%–20% of the total flour protein and contribute to the functional properties of wheat flour dough. Some alpha gliadins also contain immunodominant epitopes that trigger celiac disease, a chronic autoimmune disease that affects approximately 1% of the worldwide population. In an attempt to reduce the immunogenic potential of wheat flour from the U.S. spring wheat cultivar Butte 86, RNA interference was used to silence a subset of alpha gliadin genes encoding proteins containing celiac disease epitopes. Two of the resulting transgenic lines were analyzed in detail by quantitative two-dimensional gel electrophoresis combined with tandem mass spectrometry. Although the RNA interference construct was designed to target only some alpha gliadin genes, all alpha gliadins were effectively silenced in the transgenic plants. In addition, some off-target silencing of high molecular weight glutenin subunits was detected in both transgenic lines. Compensatory effects were not observed within other gluten protein classes. Reactivities of IgG and IgA antibodies from a cohort of patients with celiac disease toward proteins from the transgenic lines were reduced significantly relative to the nontransgenic line. Both mixing properties and SDS sedimentation volumes suggested a decrease in dough strength in the transgenic lines when compared to the control. The data suggest that it will be difficult to selectively silence specific genes within families as complex as the wheat alpha gliadins. Nonetheless, it may be possible to reduce the immunogenic potential of the flour and still retain many of the functional properties essential for the utilization of wheat.

## Introduction

The gluten proteins are a complex group of more than 50 proteins that have been intensively studied because of their important contributions to the commercial value of wheat. These proteins comprise 70%–80% of wheat flour protein, contain regions of very repetitive sequences with large proportions of glutamine (Q) and proline (P), and are responsible for the unique viscoelastic properties of the flour. The gluten proteins include glutenins, polymeric proteins that contribute elasticity to wheat flour dough, and gliadins, monomeric proteins that contribute extensibility to dough. The glutenins are composed of high molecular-weight glutenin subunits (HMW-GS) and low-molecular weight glutenin subunits (LMW-GS) that are linked by disulfide bonds whereas the gliadins consist of four distinct types of proteins, referred to as alpha, gamma, delta and omega gliadins (reviewed by Shewry, 2019). Most hexaploid wheat cultivars contain six or less HMW-GS genes. However, the numbers of genes within the complex gliadin and LMW-GS families were not known until the completion of a high-quality genome sequence from the reference wheat Chinese Spring (IWGSC, 2018) made it possible to assemble and annotate a complete set of gluten protein genes from a single hexaploid cultivar (Huo et al., 2018a; Huo et al., 2018b). In Chinese Spring, the sequences of 47 alpha gliadin, 14 gamma gliadin, five delta gliadin, 19 omega gliadin, and 17 LMW-GS genes were reported. Of these, 26 alpha, 11 gamma, two delta, and five omega gliadin, and 10 LMW-GS genes encode full-length proteins.

In addition to their role in end-use quality, the gluten proteins also trigger celiac disease (CD), a chronic autoimmune disease that affects 1.4% and 0.7% of the population worldwide, based on serology and biopsy assessments, respectively (Singh et al., 2018). CD occurs in genetically susceptible individuals that carry the human leukocyte antigen (HLA) genes DQ2 and/or DQ8 and results in damage to the lining of the intestine and malabsorption of nutrients that are manifested in a wide range of intestinal and extraintestinal symptoms (Koning, 2012). It is likely that the high glutamine and proline contents of the gluten proteins contribute to their immunogenic properties. The average Q + P content for gluten proteins in the different classes ranges from ~45% (delta gliadins) to 73% (omega gliadins). As a result, these proteins are highly resistant to proteolytic degradation within the gastrointestinal tract. The high Q + P contents of the gluten proteins also makes them good substrates for tissue transglutaminase, an enzyme in the small intestine that converts glutamine to negatively charged glutamate residues. Deamidation of gluten peptides increases their binding affinity for HLA-DQ2 and -DQ8 on antigen presenting cells, allowing them to be processed and presented to T-cells to trigger an inflammatory immune response.

Epitopes relevant to CD have been identified within all of the major classes of gluten proteins (Sollid et al., 2012). Five epitopes from alpha gliadins include the core sequences PFPQPQLPY, PYPQPQLPY, PQPQLPYPQ, FRPQQPYPQ, and QGSFQPSQQ. In some alpha gliadins, six epitopes overlap in a 33-mer protease-resistant peptide that has been found to be particularly toxic (Shan et al., 2002). Sixteen of the 26 alpha gliadins from Chinese Spring (62%) contain from one to eight CD epitopes. However, only one alpha gliadin encoded by the D genome contains the 33-mer toxic peptide. The greatest number of epitopes are found in proteins encoded by the D genome while nine of 11 alpha gliadins encoded by the B genome and one alpha gliadin encoded by the D genome do not contain any previously described epitopes (Huo et al., 2018b; Altenbach et al., 2020). Eight CD epitopes have been described in gamma gliadins, including PQQSFPQQQ, IQPQQPAQL, QQPQQPYPQ, SQPQQQFPQ, PQPQQQFPQ, PQPQQPFCQ, QQPFPQQPQ, and QQPQQPFPQ. All gamma gliadins from Chinese Spring contain from five to ten CD epitopes (Altenbach et al., 2020). Two epitopes, PFPQPQQPF and PQPQQPFPW, were identified in omega-1,2 gliadins, a subset of omega gliadins. All omega-1,2 gliadins from Chinese Spring contain these epitopes as well as multiple copies of the QQPQQPFPQ and QQPFPQQPQ gamma gliadin epitopes. Two epitopes have been described for LMW-GS, PFSQQQQPV and FSQQQQSPF. Seven of the ten LMW-GS in Chinese Spring contain from one to three of these epitopes. Finally, one epitope was identified in HMW-GS, QGYYPTSPQ (Sollid et al., 2012). In general, epitopes from alpha and omega gliadins are immunodominant (Tye-Din et al., 2010), possibly because these epitopes have a greater number of proline residues and may be more resistant to proteolytic digestion.

Currently, the only effective treatment for CD is a lifelong gluten-free diet. Thus, there is a critical need for new approaches to reduce the immunogenic potential of wheat flour. However, these studies are challenging because of the large numbers of different wheat cultivars that are grown around the world, the tremendous allelic variation in gluten protein genes among cultivars and the large number of immunogenic sequences in all of the major classes of gluten proteins. A number of studies have focused on identifying cultivars that are low in CD epitopes using DNA sequencing, quantitative protein analyses, antibody screening or targeted mass spectrometry methods (van den Broeck et al., 2010; Salentijn et al., 2013; van den Broeck et al., 2015; Prandi et al., 2016; Ribeiro et al., 2016; Malalgoda et al., 2018; Pilolli et al., 2019). Other studies have used gene silencing to reduce the amounts of immunogenic proteins in wheat flour. In a recent study, RNA interference (RNAi) was used to eliminate omega-1,2 gliadins from wheat flour (Altenbach et al., 2019). This was accomplished without notable effects on the levels of other gluten proteins in the flour. Flour from the resulting transgenic plants showed decreased reactivity to IgG and IgA antibodies from a cohort of CD patients as well as improved mixing properties relative to the nontransgenic control. A number of other studies have focused on the more complex family of alpha gliadins. Barro et al. (2015) and Becker et al. (2012) used RNAi to target all alpha gliadin genes. While the alpha gliadins were reduced significantly in both studies, there were numerous changes in the levels of other gluten proteins in the resulting transgenic lines. Sánchez-León et al. (2018) used genome editing to introduce mutations into a conserved region in the alpha gliadin genes. Alpha gliadins were reduced from 32%–82% in the resulting plants. However, reductions in alpha gliadins were accompanied by significant changes in the levels of most other types of gluten proteins. While off-target and compensatory effects on the proteome have been observed, little is known about how the proteome adjusts to significant reductions in proteins that normally comprise as much as 15%–20% of the total protein or the molecular mechanisms involved. In this study, the goal was to use RNA interference to silence only those alpha gliadin genes containing known CD epitopes with the hope that the immunoreactivity of the flour might be reduced with minimal effects on the proteome. The work highlights some of the challenges faced in experiments aimed at eliminating specific proteins within large families of gluten proteins with very similar and repetitive sequences.

## Material and Methods

### Plant Material

The U.S. hard red spring wheat *Triticum aestivum* cv. Butte 86 was used for all studies. All plant material was grown in a temperature-controlled greenhouse with daytime/nighttime temperatures of 24/17°C as described previously (Altenbach et al., 2003). Plants were supplied with water mixed with 0.6 g/l of Peters Professional 20-20-20 water-soluble fertilizer (Scotts-Sierra Horticultural Products Company, Marysville, OH) by a drip irrigation system.

### RNAi Construct and Transformation of Plants

A 608-bp DNA fragment designed to target alpha gliadins was synthesized by GenScript (Piscataway, NJ) and cloned into the vector pUC57. The 608-bp fragment consisted of a 14-bp region that included a Hpa I restriction site, a 217-bp trigger in sense orientation, a 146-bp spacer region corresponding to an intron from a wheat starch synthase gene, a 217-bp trigger in antisense orientation, and a 14-bp spacer that included a Hpa I restriction site. This plasmid was digested with Hpa I (New England Biolabs, Ipswich, MA). Following purification, the fragment was ligated into the Hpa I site of the plasmid pJL10P5 between the promoter from the HMW-GS Dy10 gene and the terminator from the HMW-GS Dx5 gene as described in Altenbach and Allen (2011). The final construct, referred to as Bazooka-pJL10P5-#6, was verified by DNA sequencing. Bazooka-pJL10P5-#6 and the plasmid pAHC20 that facilitates selection of transgenic plants with phosphinothricin (Christensen and Quail, 1996) were used to transform Butte 86 wheat plants as described in detail in Altenbach and Allen (2011). Putative transgenic plants were identified by PCR analysis using primers described in Altenbach and Allen (2011). Initial screening of gliadin fractions from grain by SDS-PAGE was also described in Altenbach and Allen (2011). Lines in which alpha gliadins were significantly down-regulated were identified and homozygous plants were selected in subsequent generations.

### Protein Extraction and Analysis by Two-Dimensional Gel Electrophoresis (2-DE)

Triplicate samples of grain from selected lines were milled into flour using a Quadrumat Senior experimental flour mill following AACCI Method 26.10.02 (AACC Int., 1988). Total proteins were extracted from the resulting flour, quantified using a modified Lowry assay and analyzed on triplicate 2-D gels using capillary tube gels with a pI range of 3 to 10 in the first dimension and NuPAGE 4%–12% BIS-Tris protein gels in the second dimension (Life Technologies, Carlsbad, CA) as described in detail in Dupont et al. (2011). Following staining with Coomassie G-250 (Sigma Aldrich, St. Louis, MO), the gels were digitized using a calibrated scanner. 2-D gels used for the analysis are shown in **Supplementary File 1**. Individual gel spots were aligned between gels and quantified using SameSpots Version 5.0 (Nonlinear Dynamics Limited, Newcastle upon Tyne, UK). Statistical analyses of spot volume data were conducted using the SameSpots software. Identifications of individual protein spots in the Butte 86 nontransgenic line were as reported in Dupont et al. (2011) or as determined in this study. Individual spots in transgenic lines were deemed to show significant changes from the nontransgenic if they had ANOVA values < 0.02 and had changes in average normalized spot volumes that were greater than 20%.

### Identification of Proteins in 2-DE Spots by Tandem Mass Spectrometry (MS/MS)

Selected protein spots from the alpha gliadin region of 2-D gels of nontransgenic and transgenic lines were excised from triplicate gels, placed in 96-well plates and digested with either chymotrypsin, thermolysin, or trypsin using a DigestPro according to the directions of the manufacturer (INTAVIS Bioanalytical Instruments AG, Cologne, Germany). The resulting samples were then analyzed using an Orbitrap Elite mass spectrometer (Thermo Scientific, San Jose, CA, USA) as described in Vensel et al. (2014). Two search engines, Mascot (www.matrixscience.com) and XTandem! (https://www.thegpm.org/TANDEM/), were used to interrogate a database of 125,400 protein sequences. The database included Triticeae sequences downloaded from NCBI on 06-18-2018 plus Chinese Spring sequences reported by Huo et al. (2018a; 2018b); Butte 86 sequences from Dupont et al. (2011) and Altenbach et al. (2011); Xiaoyan 81 sequences from Wang et al. (2017); and common mass spectrometry contaminant sequences contained in the common Repository of Adventitious Proteins (cRAP) database (ftp://ftp.thegpm.org/fasta/cRAP/crap.fasta). Data from the two searches and three enzymes were compiled and further validated using Scaffold version 4.8.9 (http://www.proteomesoftware.com/) using a protein threshold of 99%, peptide threshold of 95% and 20 ppm mass error, and a minimum of four peptides. The mass spectrometry data have been deposited to the ProteomeXchange Consortium (http://proteomecentral.proteomexchange.org) *via* the PRIDE partner repository (Perez-Riverol et al., 2019) with the dataset identifier PXD016930 and 10.6019/PXD016930. The protein that was assigned the greatest number of unique peptides was reported as the predominant protein for each spot. Proteins that were assigned at least half the number of unique peptides as the predominant protein are also reported for each spot along with the numbers of unique peptides, total spectra, and protein coverage for each. Summaries of MS/MS data from each spot are shown in **Supplementary Files 2–5**.

### Assessment of Immune Reactivity by ELISA and 2-D Immunoblot Analysis

Serum samples from a cohort of patients with celiac disease were used to assess immune reactivity toward gluten proteins from the nontransgenic and transgenic wheat lines. The celiac disease patients included twenty with elevated levels of IgG antibody to gluten [15 female, 17 white race, mean (SD) age 42.9 (18.5) years] and twenty with elevated levels of IgA antibody to gluten [13 female, 19 white race, mean (SD) age 46.7 (17.3) years]. Positivity for IgG or IgA antibody reactivity to gluten was determined as described previously (Samaroo et al., 2010). All patients were biopsy proven, diagnosed with CD according to previously described criteria (Alaedini and Green, 2005), and on a gluten-containing diet. In addition, all patients were positive for antibody reactivity to transglutaminase 2, the most sensitive and specific serologic marker of CD, determined as previously described (Lau et al., 2013). Serum samples were obtained under institutional review board-approved protocols at Columbia University. This study was approved by the Institutional Review Board of Columbia University Medical Center. Serum samples were maintained at −80°C to maintain stability.

Levels of serum IgG and IgA antibody reactivity to gluten were measured separately by enzyme-linked immunosorbent assay (ELISA) as described in Altenbach et al. (2019). All serum samples were tested in duplicate. Absorbance values were corrected for nonspecific binding by subtraction of the mean absorbance of the associated uncoated wells and corrected values were normalized according to the mean value of the positive controls on each plate. The change in immune reactivity towards the transgenic wheat lines in comparison to the nontransgenic line, as determined by ELISA, was assessed by the Wilcoxon matched-pairs test. All P values were two-sided and differences were considered statistically significant at P < 0.05. Statistical analyses were performed with Prism 8 (GraphPad) software.

IgG and IgA antibody reactivity to gluten proteins was further analyzed by two-dimensional immunoblotting as described in detail in Altenbach et al. (2019).

### Analysis of Flour End-Use Quality

End-use functionality tests were conducted at the USDA-ARS-HWWQL (Manhattan, KS) using standardized methods approved by American Association of Cereal Chemists International (AACCI). Flour protein content was determined by NIR using AACCI method 39-11.01 (AACC Int., 1985), mixing properties were determined on 10 g flour samples (14% mb) using a Mixograph (TMCO, National Mfg., Lincoln, NE) and AACCI Method 54-40.02 (AACC Int., 1995), and SDS sedimentation tests were done according to AACCI Method 56-60.01 (AACC Int., 1961). Averages and standard deviations from triplicate samples were calculated for each wheat line.

## Results

### Design of the Trigger for the RNAi Construct

**Figure 1:**
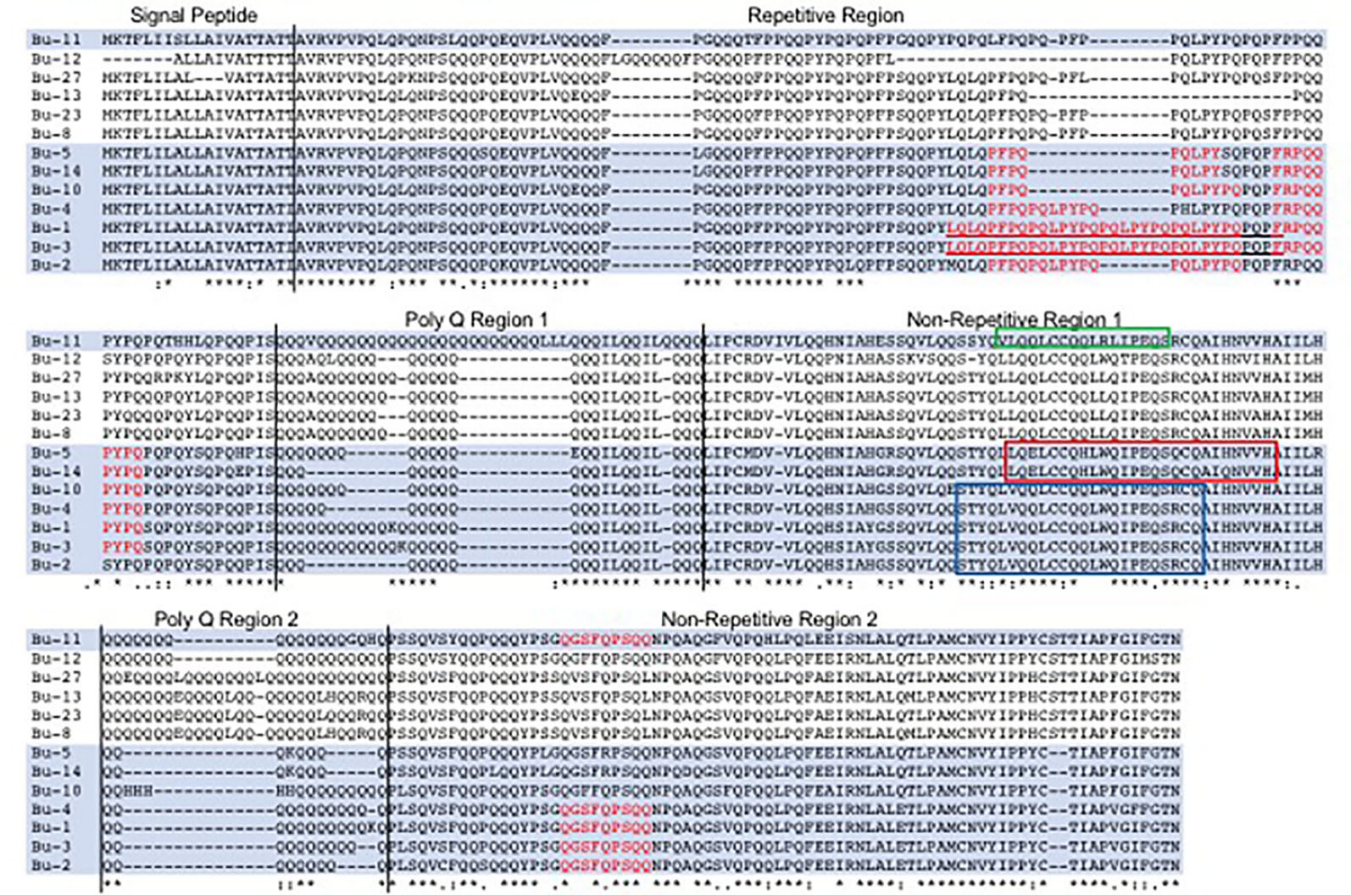
Comparison of alpha gliadins from Butte 86 and selection of target regions for the RNAi construct. Celiac disease (CD) epitope sequences within the alpha gliadins are shown in red and the 33-mer toxic peptide is underlined. Proteins that were targeted in RNAi experiments are shaded. The 74, 65, and 78 bp fragments used in the RNAi construct correspond to regions of the proteins shown in the blue, red, and green boxes, respectively. Protein sequences were derived from genes assembled from Butte 86 expressed sequence tags reported in Altenbach et al., 2010.

The 217 bp trigger for the RNAi construct consisted of three distinct fragments of 74, 65, and 78 bp that were based on sequences of 13 full-length alpha gliadin coding regions assembled from Butte 86 expressed sequence tags (ESTs) available at the time the study was initiated (Altenbach et al., 2010). All three target fragments encode a portion of the first nonrepetitive region of the alpha gliadin that lies between the two poly Q regions (**Figure 1**). Target 1, a 74-bp fragment with the sequence AAAGTACTTACCAGCTGGTGCAACAATTGTGTTGTCAGCAGCTGTGGCAGATCCCCGAGCAGTCGCGGTGCCAA, was a perfect match with alpha gliadins Bu-1, Bu-2, Bu-3, Bu-4, and Bu-10 (**Table 1**). These genes are likely from the D genome and encode proteins containing from three to eight CD epitopes. This fragment also had 25 bp of identity with Bu-11. Target 2, a 65-bp fragment with the sequence TTGCAAGAATTGTGTTGTCAGCACCTATGGCAGATCCCTGAGCAGTCGCAGTGCCAGGCCATCCA, was a perfect match with Bu-5 and Bu-14, likely from the A genome and encoding proteins containing two CD epitopes, while Target 3, a 78-bp fragment with the sequence AAGTATTGCAGCAAAGTAGTTACCAAGTGTTGCAACAATTATGTTGTCAGCAGCTGCGGCTGATCCCCGAGCAGTCGC, was a perfect match with Bu-11 encoding a protein with one CD epitope. The 78-bp fragment also had 25 bp of identity with Bu-12 that encodes a protein without any epitopes. Seven alpha gliadin genes from Butte 86 had 19 or less bp of identity with Target 1 while 11 genes had 19 or less bp of identity with either Target 2 or Target 3. None of the targets had identities greater than 19 bp with Bu-8, Bu-13, Bu-23, or Bu-27, all of which are likely to be from the B genome in Butte 86 and encode proteins devoid of CD epitopes.

**Table 1:** Identities of target sequences used in trigger of RNAi construct to alpha gliadin genes from Butte 86.

Alpha gliadin gene	# CD epitopes in protein	Longest region of identity (bp)		
		Target 1^1^	Target 2^2^	Target 3^3^
Bu-1	8	74	16	17
Bu-2	4	74	16	17
Bu-3	8	74	16	17
Bu-4	4	74	16	17
Bu-5	2	17	65	<16
Bu-8	0	19	19	<16
Bu-10	3	74	16	17
Bu-11	1	25	<16	78
Bu-12	0	<16	<16	25
Bu-13	0	19	19	<16
Bu-14	2	16	65	<16
Bu-23	0	19	19	<16
Bu-27	0	19	19	<16

^1^74 bp target sequence is AAAGTACTTACCAGCTGGTGCAACAATTGTGTTGTCAGCAGCTGTGGCAGATCCCCGAGCAGTCGCGGTGCCAA.

^2^65 bp target sequence is TTGCAAGAATTGTGTTGTCAGCACCTATGGCAGATCCCTGAGCAGTCGCAGTGCCAGGCCATCCA.

^3^78 bp target sequence is AAGTATTGCAGCAAAGTAGTTACCAAGTGTTGCAACAATTATGTTGTCAGCAGCTGCGGCTGATCCCCGAGCAGTCGC.

Because the collection of alpha gliadin sequences from Butte 86 is incomplete, the specificities of the target sequences also were assessed using the complete set of 26 full-length alpha gliadin genes that was recently reported from the reference wheat Chinese Spring (Huo et al., 2018b) (**Table 2**). Five alpha gliadins encoded by the D genome (CS-D4, CS-D5, CS-D6, CS-D8, CS-D9) were perfect matches with Target 1, four encoded by the A genome (CS-A4, CS-A5, CS-A9, CS-A10) were perfect matches with Target 2, and one encoded by the A genome (CS-A2) was a perfect match with Target 3. In addition, five genes had regions of identities between 25 and 56 bp with Target 1, one gene had a 53-bp region of identity with Target 2 and nine genes had regions of identities between 21 and 29 bp with Target 3. Only four of the 26 alpha gliadins in Chinese Spring did not have identities greater than 19 bp with any of the targeting regions. Three of these are from the B genome (CS-B7, CS-B8, CS-B9) and do not contain CD epitopes and one is from the A genome (CS-A1) and contains a single epitope. A BLASTn search also revealed that there were no regions of identity 16 bp or greater with any gamma, delta, or omega gliadins, LMW-GS, or HMW-GS from Chinese Spring, suggesting that the RNAi construct should target only alpha gliadins.

**Table 2:** Identities of target sequences used in trigger of RNAi construct to alpha gliadin genes from Chinese Spring.

Alpha gliadin gene	# CD epitopes in protein	Longest region of identity (bp)		
		Target 1^1^	Target 2^2^	Target 3^3^
CS-A1	1	<16	<16	<16
CS-A2	1	25	<16	78
CS-A4	2	17	65	<16
CS-A5	2	17	65	<16
CS-A6	2	17	62	<16
CS-A8	2	17	53	<16
CS-A9	1	17	65	<16
CS-A10	2	16	65	<16
CS-B3	1	32	<16	22
CS-B7	0	19	19	<16
CS-B8	0	19	19	<16
CS-B9	0	19	19	<16
CS-B11	0	<16	<16	21
CS-B14	0	<16	<16	25
CS-B15	0	<16	<16	25
CS-B16	0	<16	<16	25
CS-B17	0	<16	<16	25
CS-B18	0	<16	<16	25
CS-B25	1	56	16	17
CS-D1	1	45	<16	29
CS-D4	3	74	16	17
CS-D5	8	74	16	17
CS-D6	5	74	16	17
CS-D8	6	74	16	17
CS-D9	6	74	16	17
CS-D12	0	27	<16	23

^1^74 bp target sequence is AAAGTACTTACCAGCTGGTGCAACAATTGTGTTGTCAGCAGCTGTGGCAGATCCCCGAGCAGTCGCGGTGCCAA.

^2^65 bp target sequence is TTGCAAGAATTGTGTTGTCAGCACCTATGGCAGATCCCTGAGCAGTCGCAGTGCCAGGCCATCCA.

^3^78 bp target sequence is AAGTATTGCAGCAAAGTAGTTACCAAGTGTTGCAACAATTATGTTGTCAGCAGCTGCGGCTGATCCCCGAGCAGTCGC.

### Analysis of Flour Proteins From Transgenic Lines

**Figure 2:**
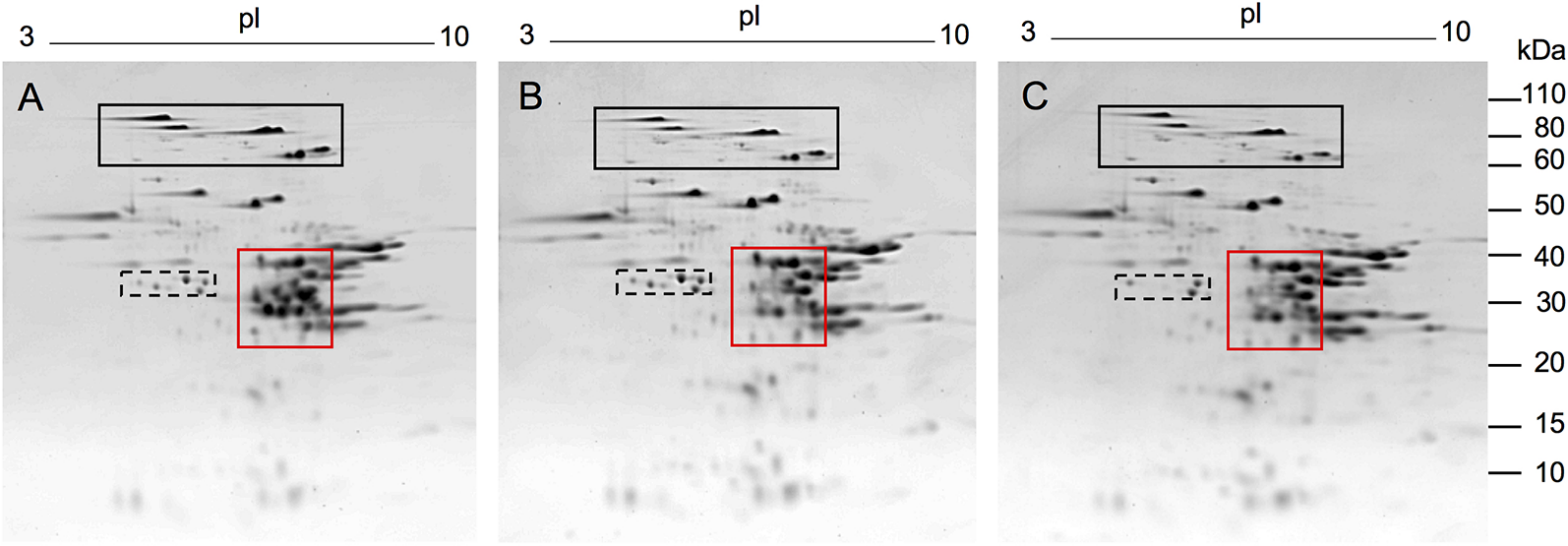
2-DE analysis of total flour proteins from the nontransgenic **(A)** and transgenic lines 124j **(B)** and 658-5 **(C)**. MW are indicated on the right and the pI range is shown above each gel. Red boxes show regions of gels containing alpha gliadins. These regions were enlarged in **Figure 3**. The black and black dashed boxes show regions of gels containing HMW-GS and serpins, respectively. Proteins were identified by MS/MS in Dupont et al. (2011).

**Figure 3:**
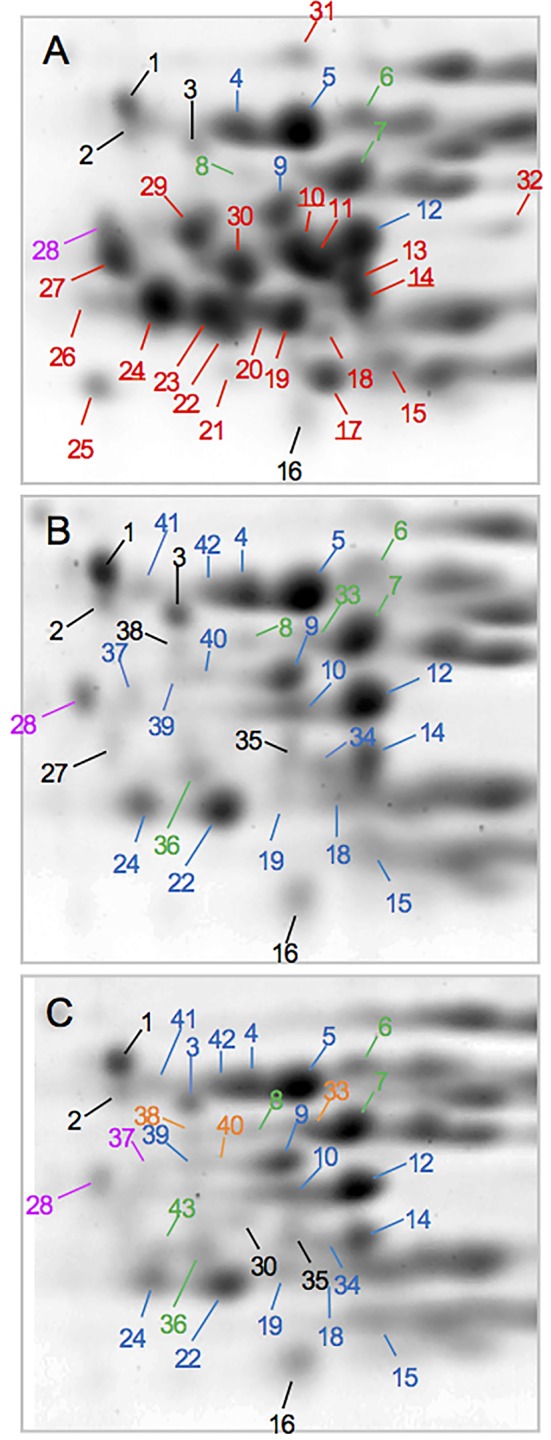
Regions of 2-D gels containing alpha gliadins from the nontransgenic **(A)** and transgenic lines 124j **(B)** and 658-5 **(C)**. Spots in which the predominant protein was identified as an alpha gliadin are shown in red, gamma gliadin in blue, delta gliadin in magenta, and LMW-GS in green. Spots with underlined numbers in panel A contained a gamma gliadin in addition to an alpha gliadin. Spots shown in black were identified as nongluten proteins while those labeled in orange did not yield identifications.

Following transformation of Butte 86 plants and initial DNA and protein analyses, two homozygous transgenic lines showing altered alpha gliadin profiles in SDS-PAGE were selected for detailed analysis by quantitative 2-DE. Total protein profiles are shown in **Figure 2** for transgenic lines SA35a-124j and SA39b-658-5, referred to as 124j and 658-5, respectively. While most alpha gliadins in the nontransgenic flour are found within the red box shown in Panel A, this region also contains some gamma gliadins, LMW-GS and the nongluten storage proteins called triticins. There are notable changes in this region of the gel for the two transgenic lines shown in panels B and C. As can be seen in the enlarged alpha gliadin regions in **Figure 3**, some spots present in the nontransgenic line are missing in the transgenic lines and some are significantly reduced. Additionally, in a number of cases the suppression of a major spot found in the nontransgenic line revealed the presence of several minor spots in the transgenic lines. To investigate this further, 30 spots in the alpha gliadin region as well as two spots that lie outside of this region and were previously identified as alpha gliadins (spots 31, 32) were excised from triplicate 2-D gels of the nontransgenic Butte 86. Spots in corresponding positions in the transgenic lines as well as new spots uncovered in the transgenic lines were also excised from triplicate gels (**Figure 3**). Following digestion with either chymotrypsin, thermolysin, or trypsin, all spots were analyzed by MS/MS.

Not surprisingly, the identifications were complex (**Table 3**, **Supplementary Files 2–5**). In the nontransgenic line, the predominant proteins in 20 spots were alpha gliadins (10, 11, 13, 14, 15, 17–27, 29–32). For these spots, the MS sequence coverage ranged from 29 to 89% with an average of 63%. The predominant proteins in four spots were gamma gliadins (4, 5, 9, 12) while the predominant proteins in three spots were LMW-GS (6, 7, 8). Three of the alpha gliadin spots also contained other alpha gliadins (20, 24, 26) while five also contained gamma gliadins (10, 14, 17, 22, 24). In addition, one spot contained a delta gliadin mixed with an alpha gliadin (28), one contained an avenin-like protein (16), and three contained triticins (1, 2, 3), two of which were mixed with gamma gliadins (2, 3).

**Table 3:** Predominant proteins identified by MS/MS in 2-DE protein spots from the alpha gliadin region of nontransgenic Butte 86 and transgenic lines 124j and 658-5. The positions of the spots are shown in **Figure 3**. MS data can be found in **Supplementary Files 2–5**.

Line	Spot #	Predominant Protein	Accession^1^	# Unique Peptides	# Spectra	% Coverage
Butte 86	1	triticin	ACB41345	7	14	14
Butte 86	2	triticin^2^	AAB27108	21	39	291
Butte 86	3	triticin^2^	EMS60011	20	34	26
Butte 86	4	gamma gliadin	Bu-Gamma5	49	121	68
Butte 86	5	gamma gliadin	Bu-Gamma5	79	181	84
Butte 86	6	LMW-GS (s-type)	CS-LMW-D1	50	109	75
Butte 86	7	LMW-GS (m-type)	Bu-LMW-7	87	210	85
Butte 86	8	LMW-GS (m-type)	AVY03606 (Bu-LMW7)	13	23	43
Butte 86	9	gamma gliadin	EMS45054 (Bu-Gamma1)	40	80	57
Butte 86	10	alpha gliadin^2^	X-Alpha18 (Bu-Alpha23)	30	67	52
Butte 86	11	alpha gliadin	Bu-Alpha23	67	145	77
Butte 86	12	gamma gliadin	Bu-Gamma2	61	165	87
Butte 86	13	alpha gliadin	SCW25764 (Bu-Alpha 1)	47	100	73
Butte 86	14	alpha gliadin^2^	AKC91252 (Bu-Alpha 1)	26	53	58
Butte 86	15	alpha gliadin	Bu-Alpha2	43	89	70
Butte 86	16	avenin-like protein	Bu-farinin-2	16	34	49
Butte 86	17	alpha gliadin^2^	Bu-Alpha5	44	120	81
Butte 86	18	alpha gliadin	SCW25769 (Bu-Alpha10)	32	53	59
Butte 86	19	alpha gliadin	Bu-Alpha10	35	73	57
Butte 86	20	alpha gliadin^3^	CS-Alpha-B11 (Bu-Alpha12)	45	118	71
Butte 86	21	alpha gliadin	Bu-Alpha5	5	9	29
Butte 86	22	alpha gliadin^2^	Bu-Alpha4	44	89	77
Butte 86	23	alpha gliadin	Bu-Alpha4	82	159	89
Butte 86	24	alpha gliadin^2,3^	AKC91122	49	122	76
Butte 86	25	alpha gliadin	Bu-Alpha14	14	22	47
Butte 86	26	alpha gliadin^3^	AKC91122	8	12	42
Butte 86	27	alpha gliadin	CS-Alpha-B16/B17	18	36	40
Butte 86	28	delta gliadin^3^	CS-delta-D1	11	22	33
Butte 86	29	alpha gliadin	CAY54134 (Bu-Alpha12)	51	120	74
Butte 86	30	alpha gliadin	Bu-Alpha3	83	165	86
Butte 86	31	alpha gliadin	CS-Alpha-B3	16	32	39
Butte 86	32	alpha gliadin	Bu-Alpha27	33	70	60
transgenic 124j	1	triticin	ABC41345	48	102	46
transgenic 124j	2	triticin	ABC41345	25	57	37
transgenic 124j	3	triticin^2^	EMS60011	40	78	36
transgenic 124j	4	gamma gliadin	Bu-Gamma5	37	80	72
transgenic 124j	5	gamma gliadin	Bu-Gamma5	77	181	81
transgenic 124j	6	LMW-GS (s-type)	CS-LMW-D1	44	80	79
transgenic 124j	7	LMW-GS (m-type)	ALN96387 (Bu-LMW-7)	52	118	71
transgenic 124j	8	LMW-GS (m-type)	Bu-LMW-7	16	27	39
transgenic 124j	9	gamma gliadin	Bu-Gamma1	29	59	52
transgenic 124j	10	gamma gliadin	Bu-Gamma2	40	91	81
transgenic 124j	12	gamma gliadin	Bu-Gamma2	49	139	83
transgenic 124j	14	gamma gliadin	Bu-Gamma3	26	52	59
transgenic 124j	15	gamma gliadin^2^	Bu-Gamma7	18	39	39
transgenic 124j	16	avenin-like protein	AEW43832	10	24	29
transgenic 124j	18	gamma gliadin^2^	Bu-Gamma4	19	36	57
transgenic 124j	19	gamma gliadin	ACI04093	15	26	34
transgenic 124j	22	gamma gliadin	CS-Gamma-D3	45	104	74
transgenic 124j	24	gamma gliadin	CS-Gamma-D3	40	76	71
transgenic 124j	27	glyceraldehyde-3-phosphate dehydrogenase^6^	ANW11922	17	32	40
transgenic 124j	28	delta gliadin	CS-delta-D1	30	59	59
transgenic 124j	33	LMW-GS (m-type)^2^	Bu-LMW-7	19	29	39
transgenic 124j	34	gamma gliadin^4^	Bu-Gamma3	16	31	37
transgenic 124j	35	glyceraldehyde-3-phosphate dehydrogenase^2^	ALE18233	14	24	36
transgenic 124j	36	LMW-GS (m-type)	CS-LMW-D7	35	109	59
transgenic 124j	37	gamma gliadin^5,6^	EMS45054	7	11	15
transgenic 124j	38	triticin	EMS60011	12	19	25
transgenic 124j	39	gamma gliadin	EMS45054	6	14	15
transgenic 124j	40	gamma gliadin	Bu-Gamma8	14	30	32
transgenic 124j	41	gamma gliadin	Bu-Gamma5	17	34	37
transgenic 124j	42	gamma gliadin	Bu-Gamma5	19	39	39
transgenic 658-5	1	triticin	ACB41345	28	69	43
transgenic 658-5	2	triticin	ACB41345	22	60	42
transgenic 658-5	3	gamma gliadin^6^	Bu-Gamma5	16	27	33
transgenic 658-5	4	gamma gliadin	Bu-Gamma5	26	48	52
transgenic 658-5	5	gamma gliadin	Bu-Gamma5	47	140	69
transgenic 658-5	6	LMW-GS (s-type)	CS-LMW-D1	23	40	61
transgenic 658-5	7	LMW-GS (m-type)	Bu-LMW7	46	115	66
transgenic 658-5	8	LMW-GS (m-type)	Bu-LMW7	21	36	47
transgenic 658-5	9	gamma gliadin	EMS45054 (Bu-Gamma1)	17	33	44
transgenic 658-5	10	gamma gliadin	AGO17694 (Bu-Gamma2)	22	44	60
transgenic 658-5	12	gamma gliadin	AAF42989 (Bu-Gamma2)	42	86	66
transgenic 658-5	14	gamma gliadin	Bu-Gamma3	18	30	54
transgenic 658-5	15	gamma gliadin	ACJ03439 (Bu-Gamma7)	12	27	41
transgenic 658-5	16	avenin-like protein	AEW43832	20	47	57
transgenic 658-5	18	gamma gliadin^2^	ATD83912	20	37	46
transgenic 658-5	19	gamma gliadin^2,3,6^	ACI04093	7	22	24
transgenic 658-5	22	gamma gliadin	BAN29066	40	94	77
transgenic 658-5	24	gamma gliadin	CS-Gamma-D3	18	32	51
transgenic 658-5	28	delta gliadin	CS-delta-D1	14	27	34
transgenic 658-5	30	glyceraldehyde-3-phosphate dehydrogenase^2,3,6^	ANW11921	8	15	30
transgenic 658-5	33	no ID				
transgenic 658-5	34	gamma gliadin	Bu-gamma3	7	14	24
transgenic 658-5	35	glyceraldehyde-3-phosphate dehydrogenase	ANW11922	16	32	47
transgenic 658-5	36	LMW-GS (m-type)^6^	AFL55408	7	18	23
transgenic 658-5	37	delta gliadin^2^	CS-delta-D1	6	14	24
transgenic 658-5	38	no ID				
transgenic 658-5	39	gamma gliadin^6^	Bu-Gamma8	5	11	16
transgenic 658-5	40	no ID				
transgenic 658-5	41	gamma gliadin^6^	Bu-Gamma5	11	22	35
transgenic 658-5	42	gamma gliadin	Bu-Gamma5	27	59	60
transgenic 658-5	43	LMW-GS (m-type)	CS-LMW-D7	5	9	18

^1^Protein accessions from Butte 86 begin with Bu and are reported in Dupont et al. (2011), proteins from Chinese Spring begin with CS and are reported in Huo et al. (2018a; 2018b), and proteins from Xioayan 81 begin with X and are reported in Wang et al. (2018). All other accessions are from NCBI. Proteins from Butte 86 that are very similar to the protein identified by Scaffold are shown in parentheses.

^2^Spot also contains gamma gliadin.

^3^Spot also contains other alpha gliadins.

^4^Spot also contains LMW-GS.

^5^Spot also contains delta gliadin.

^6^Spot also contains non-gluten protein.

Twelve of the spots identified as alpha gliadins in the nontransgenic line were missing in transgenic line 124j (11, 13, 17, 20, 21, 23, 25, 26, 29, 30, 31, 32) while seven spots identified as alpha gliadins in the nontransgenic line were identified as gamma gliadins (10, 14, 15, 18, 19, 22, 24) in 124j. A delta gliadin was the only protein identified in spot 28 in the transgenic line. Of the ten other minor spots that were uncovered in the transgenic line, six were gamma gliadins (34, 37, 39–42), two were LMW-GS (33, 36), one was triticin (38) and one was glyceraldehye-3-phosphate dehydrogenase (35). No alpha gliadins were identified in 124j in the analysis.

Similarly, twelve spots identified as alpha gliadins in the nontransgenic line were missing in transgenic line 658-5 (11, 13, 17, 20, 21, 22, 25, 26, 27, 29, 31, 32), and the same seven spots that were identified as alpha gliadins in the nontransgenic line but gamma gliadins in 124j were also identified as gamma gliadins in 658-5 (10, 14, 15, 18, 19, 22, 24). Spot 28 in 658-5 contained only a delta gliadin and the identities of the minor spots uncovered in 658-5 were similar to those from 124j (**Table 3**). Alpha gliadins were identified as one of several components of two minor spots (19, 30) in 658-5.

### Quantitative Analysis of Proteins in Nontransgenic and Transgenic Lines

Most spots in which the predominant proteins were alpha gliadins in Butte 86 showed significantly reduced volumes in the transgenic lines (73% of alpha gliadin spots in 124j and 79% of alpha gliadin spots in 658-5) (**Supplementary Files 6, 7**). Decreases ranged from 25.2% to 83.9% with an average reduction of 56.6% in 124j, and 28.5% to 67.0% with an average reduction of 48.8% in 658-5. Surprisingly, a large percentage of the spots that were identified as HMW-GS also showed significantly reduced volumes (71% of HMW-GS spots in 124j and 76% of HMW-GS spots in 658-5), although the reductions were generally much smaller for the HMW-GS than the alpha gliadins. Changes in a few spots identified as omega gliadins (four of 16 spots in 124j and one of 15 in 658-5) and LMW-GS (three of 22 spots in 124j and four of 22 spots in 658-5) also were observed. In 124j, increases were observed in the volumes of spots containing a variety of nongluten proteins, including purinins, triticins, globulins, serpins and alpha amylase inhibitors (AAI) (**Supplementary File 6**) while decreases in a number of serpins were observed in 658-5 as well as increases among purinins, globulins and a few AAI (**Supplementary File 7**). It is notable that some of these proteins, including purinins, globulins, serpins and AAI may also be involved in wheat-related pathologies.

A number of adjustments were made to the normalized spot volume data for 2-DE spots containing either alpha or gamma gliadins (**Supplementary Files 6**, **7**). First, for the five Butte 86 spots in which both alpha and gamma gliadins were identified (10, 14, 17, 22, 24), average spot volumes for Butte 86 were divided among the two protein types according to the percentage of unique peptides that were obtained for each type as detailed in **Supplementary Files 6**, **7**. Second, in the transgenic lines, the average normalized volumes were assigned to gamma gliadins since gamma gliadins were the only proteins identified in these spots in these lines. Additionally, in cases where spots were identified as alpha gliadins in Butte 86 but as gamma gliadins in the transgenic lines (15, 18, 19, 22), the entire spot volume was assigned to alpha gliadins in Butte 86, but to gamma gliadins in the transgenic lines. Additionally, spot volume data for spot 28 was divided between alpha and delta gliadins in Butte 86, but assigned to delta gliadins for the transgenic lines and spot volume data for spots 27 and 30 were assigned to alpha gliadins for Butte 86, but to the nongluten protein group for lines 124j and 658-5, respectively, since the spots were identified as glyceraldehyde-3-phosphate dehydrogenase in the transgenic lines.

Overall, decreases in the amounts of alpha gliadins of 70.4% and 66.1% and decreases in the amounts of HMW-GS of 26% and 28.8% were observed in transgenic lines 124j and 658-5, respectively (**Table 4**). Among the HMW-GS, significant decreases were noted for all subunits except Ax2* with the greatest decreases noted for HMW-GS Dy10 (34.7% and 42.2% for 124j and 658-5, respectively) (**Supplementary Files 6**, **7**). Serpins showed a 30.5% decrease in 658-5 but a slight increase in 124j. Small increases in the amounts of purinins were also noted in both transgenic lines as well as small increases in the amounts of some of the other nongluten proteins.

**Table 4:** Changes in amounts of different classes of flour proteins in transgenic lines 124j and 658-5 relative to the nontransgenic.

	% Change	
	SA35-124j	SA39b-658-5
alpha gliadins	−70.4	−66.1
gamma gliadins	15.0	1.7
omega gliadins	8.1	−3.3
delta gliadins	26.8	4.4
HMW-GS	−26.0	−28.8
LMW-GS	−2.6	−4.4
purinins	32.0	26.4
farinins	−0.3	−0.8
triticins	9.2	−2.3
globulins	21.9	15.2
serpins	21.9	−30.5
AAI	11.8	3.6
other nongluten proteins	21.9	27.0

The ratio of glutenin to gliadin in the nontransgenic line was 0.94 to 0.96 while that of both transgenics was slightly higher, 1.04 for 124j and 1.06 for 658-5. The ratio of HMW-GS to LMW-GS was 0.64 and 0.67 in Butte 86, but 0.49 and 0.50 in 124j and 658-5, respectively (**Supplementary Files 5** and **6**).

### Immunogenic Potential of Transgenic Lines

**Figure 4:**
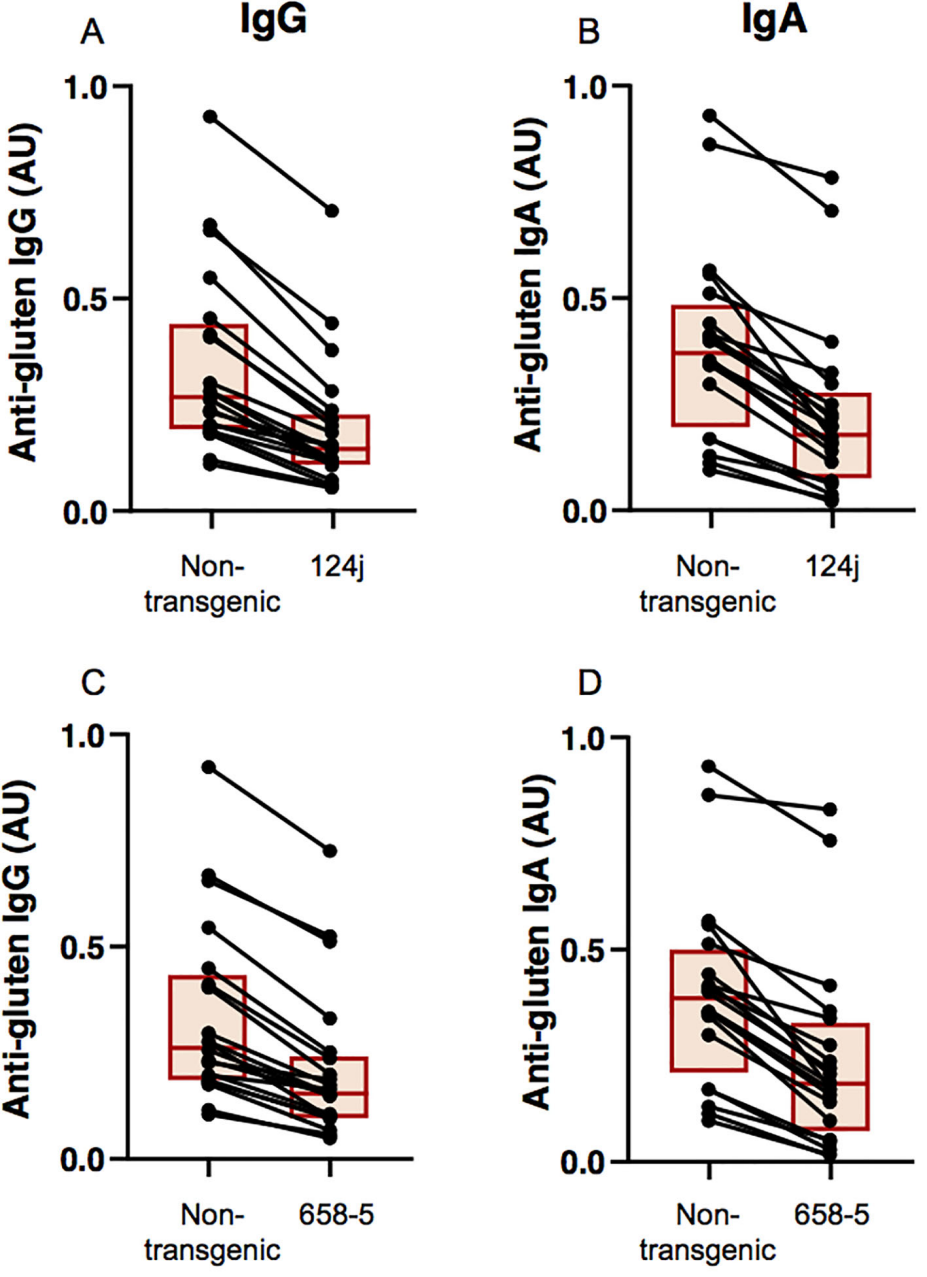
Measurement of celiac disease antibody reactivity to nontransgenic and transgenic wheat gluten proteins. Levels of antibody reactivity towards gluten proteins from nontransgenic and transgenic 124j **(A**, **B)** and 658-5 **(C**, **D)** plants are shown for each of the 20 antigluten IgG-positive **(A**, **C)** and 20 antigluten IgA-positive **(B**, **D)** celiac disease patients, as determined by ELISA. Each individual is represented by a dot and the two points corresponding to the same individual are connected by a line. Each box indicates the 25th–75th percentiles of distribution, with the horizontal line inside the box representing the median.

The immunogenic potential of the transgenic lines relative to the nontransgenic line was assessed by comparing the reactivity of antibodies from patients with biopsy-confirmed cases of CD towards flour proteins from the nontransgenic and transgenic lines. Levels of serum IgG and IgA reactivity were significantly reduced for the transgenic lines when compared to the nontransgenic line as determined by ELISA (p< 0.0001 for all comparisons) (**Figure 4**). All patients in the study had lower IgG and IgA reactivities to the transgenic lines than to the nontransgenic line, although differences were small for some patients. Reductions were similar for both transgenic lines.

**Figure 5:**
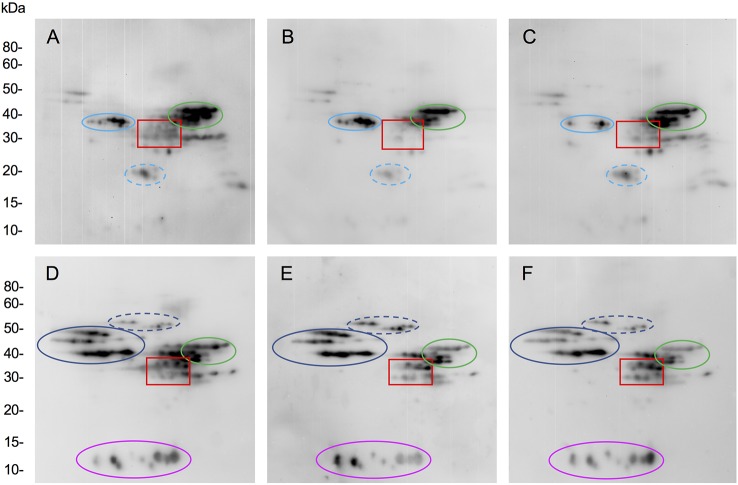
Assessment of the molecular specificity of immune reactivity towards nontransgenic and transgenic wheat lines. Immunoblots show IgG **(A–C)** and IgA **(D–F)** antibody reactivity in representative celiac disease patients towards two dimensionally separated total flour proteins from nontransgenic **(A**, **D)** and transgenic plants 124j **(B**, **E)** and 658-5 **(C**, **F)**. Red boxes show the alpha gliadin regions. In panels **(A–C)**, the positions of serpins, low-molecular weight glutenin subunits (LMW-GS) and purinins are shown in solid light blue, green, and dashed light blue ovals. In panels **(D–F)**, the positions of omega-1,2 gliadins, omega-5 gliadins, LMW-GS, and AAI are shown in solid blue, dashed blue, green, and magenta ovals. MW are indicated on the left.

The molecular specificity of immune reactivity to gluten proteins in the transgenic lines was examined by two-dimensional immunoblotting (**Figure 5**). The observed decrease in levels of IgG and IgA antigluten antibodies to transgenic lines as determined by ELISA was confirmed to be due to a reduction in antibody binding to alpha gliadins. Generally, IgG and IgA antibodies from patients reacted with a number of proteins in addition to the alpha gliadins and the profiles of reactivity varied among patients. For the representative case shown in **Figure 5A**, IgG antibodies from one patient exhibited reactivity with alpha gliadins, LMW-GS, serpins and purinins in the nontransgenic line, while in another case shown in **Figure 5D**, IgA antibodies showed reactivity with alpha gliadins, omega-1,2 gliadins, some LMW-GS and gamma gliadins, omega-5 gliadins and AAI proteins. In both cases, the overall observed reduction in IgG and IgA antibody reactivity toward the transgenic lines was attributable to a reduction in reactivity to alpha gliadin proteins.

### End-Use Quality Analysis of Transgenic Lines

**Figure 6:**
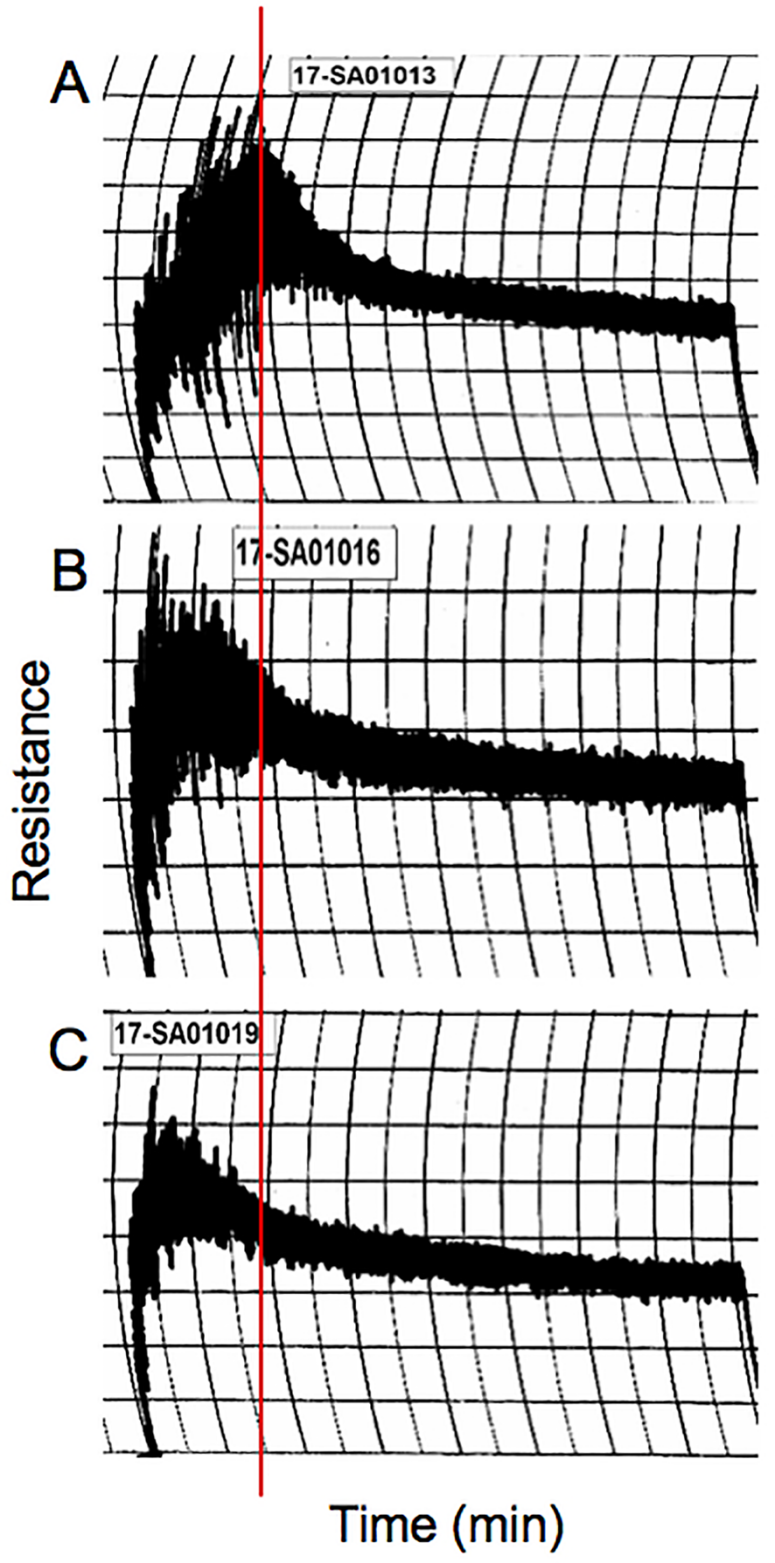
10 g mixograph curves produced using flour from nontransgenic **(A)** and transgenic lines 124j **(B)** and 658-5 **(C)**. The red line shows the position of the peak mixing time in the nontransgenic line.

Sufficient quantities of grain from the nontransgenic and transgenic lines were produced in the greenhouse for end-use quality testing using methods that are commonly utilized to assess breeding lines in the U.S. The average kernel weight of transgenic line 124j was similar to that of the nontransgenic control, 42.7 mg +/−0.7 versus 42.8 mg +/−2.2 while that of 658-5 was 24.6% less, 32.3 mg +/−1.6. Nonetheless, grain protein contents (%) were similar in the transgenic lines and the nontransgenic control while flour protein contents (%) were somewhat less than the control in the two transgenic lines (**Table 5**). The overall shapes of the mixing curves generated with a 10-g mixograph were similarly poor in both the transgenic and the nontransgenic lines. However, both transgenic lines had shorter mix times and peak heights than the nontransgenic control (**Figure 6**, **Table 5**). Water absorption decreased in flour from both transgenic lines relative to the control while mixing tolerance was poor in all lines. The SDS sedimentation volumes for the two transgenic lines were 69.2% and 59.6% less than that of the nontransgenic line, 21.1 ml/g for 124j and 27.7 ml/g for 658-5 as opposed to 68.6 ml/g for Butte 86 (**Table 5**).

**Table 5:** End-use quality data from nontransgenic Butte 86 and transgenic lines 124j and 658-5.

Line	Grain protein (%)	Flour protein^1^ (%)	Water Absorption^1^ (%)	Mix time (min)	Mix tolerance^2^	SDS sedimentation volume (ml/g)
Butte 86^3^	19.7 (0.2)	17.4 (0.5)	73.2 (1.3)	2.0 (0.4)	0.3 (0.6)	68.6 (2.17)
124j^3^	19.2 (0.7)	15.3 (0.6)	59.8 (0.8)	0.7 (0.1)	0.0 (0.0)	21.1 (2.71)
658-5^3^	19.2 (0.1)	15.5 (0.1)	64.7 (1.7)	1.1 (0.1)	0.3 (0.6)	27.7 (3.55)

^1^Based on 14% moisture.

^2^Recorded on a 0–6 scale with 6 having the greatest tolerance.

^3^Averages and (standard deviations) from flour samples from three biological replicates are reported.

## Discussion

Given the large numbers of alpha gliadin genes as well as the high similarities and repetitiveness of their sequences, it can be difficult to find regions that are unique for specific genes. This is even more challenging when not all of the gene sequences from the cultivar of interest are known. In this study, only 13 alpha gliadin gene sequences were available from Butte 86 when the RNAi construct was designed. Based on the finding that Chinese Spring contains 26 genes encoding full-length alpha gliadins, it is likely that the available sequences account for only one half of the total number of gene sequences expressed in this cultivar. Nonetheless, we focused on the regions of the 13 genes encoding the first nonrepetitive portion of the proteins with the aim of designing an RNAi construct that would target only those alpha gliadins containing CD epitopes. The average percentage of Q+P in this region is much lower than that of the N-terminal and repetitive regions that were used as targets in the RNAi constructs of Barro et al. (2015) and Becker et al. (2012). In fact, among the 26 alpha gliadins from Chinese Spring, the average percentage of Q+P in this region was 24.9% as opposed to 69.2% for the N-terminal and repetitive regions. Thus, it might be expected that the construct would be less likely to silence nontargeted genes or genes in other gluten protein families. To further decrease the likelihood of off-target effects, we also selected a trigger for the RNAi construct that consisted of fragments of contiguous sequence that were less than 78 bp in length. In comparison, the triggers in the Barro et al. (2015) and Becker et al. (2012) constructs contained 377 and 313 bp, respectively. Despite these efforts, the desired specificity was not achieved in our experiments. Rather, all alpha gliadin genes were silenced effectively in the transgenic plants, demonstrating that regions of identity less than 20 bp between the trigger and the target gene can result in silencing of gluten protein genes. This is consistent with reports that triggers with as few as 14 nucleotides of contiguous sequence complementarity sometimes result in suppression (Senthil-Kumar and Mysore, 2011).

Even more surprising is the partial suppression of HMW-GS genes that was observed in the transgenic lines given that there is little identity between the trigger region and HMW-GS sequences. However, the RNAi construct did include the promoter and 5' untranslated region from the Dy10 HMW-GS gene and the 3' untranslated region and terminator from the Dx5 HMW-GS gene. It is thus possible that the partial reduction of HMW-GS is due to cosuppression of the HMW-GS genes as a result of homology dependent gene silencing. Silencing of endogenous genes has been reported when HMW-GS transgenes were introduced into transgenic plants (Alvarez et al., 2000). However, it should be noted that the same HMW-GS promoter and terminator regions were included in RNAi constructs that targeted the omega-5 and omega-1,2 gliadin genes in other studies. Partial decreases in HMW-GS were observed in one of four transgenic lines in which the omega-5 gliadins were suppressed (Altenbach et al., 2014a), but not in lines in which the omega-1,2 gliadins were suppressed (Altenbach et al., 2019), suggesting that the copy number and/or site of insertion may also be important. Nonetheless, as a caution it may be wise to use avoid using regulatory regions derived from wheat gluten protein genes in future studies.

Surprisingly, there was little change in the levels of other gluten proteins in the transgenic lines as determined by quantitative 2-DE. Rather, the reductions in alpha gliadins and HMW-GS were compensated partially by increases in a number of nongluten proteins. Thus, it is possible to eliminate an entire group of gluten proteins without compensatory effects on other gluten protein classes. In contrast, Barro et al. (2015) stated that decreases in alpha gliadins were offset mostly by increases in HMW-GS while Becker et al. (2012) reported that lines that had the largest decreases in alpha gliadins showed increases in omega and gamma gliadins, HMW-GS, and albumins/globulins. Becker et al. (2012) also observed off-target suppression of LMW-GS in some of their lines. They hypothesized that some of the reductions in LMW-GS might be due to the suppression of alpha gliadins that contain an extra cysteine and thus are linked into the glutenin polymer. However, no evidence was provided to support this notion. Undoubtedly, both off-target and compensatory effects of RNA interference are complicated, particularly among gene families as complex as the gliadins and glutenins, and therefore require further study. Indeed, one study in which omega-5 gliadins genes were silenced by RNAi yielded transgenic lines in which there were minimal off-target or compensatory effects on the proteome as well as lines that showed notable changes in protein groups other than those targeted (Altenbach et al., 2014a). This study also employed quantitative 2-DE.

Both transgenic lines exhibited a significant reduction in binding to IgG and IgA antibodies from CD patients in comparison to the nontransgenic wheat, suggesting a decreased immunogenic potential. The reductions were similar in the two lines, as expected from their similarities in protein composition. However, the reductions were less substantive than what was observed in a previous study with transgenic plants missing the omega-1,2 gliadins (Altenbach et al., 2019), demonstrating the complexity and broad range of immunogenic gluten proteins in the context of celiac disease. Clearly, additional studies that address potential T cell reactivity of flour proteins from the transgenic lines are warranted. Considering the fact that the alpha gliadins contain known T cell epitopes and that most of these epitopes are located within the B cell epitope sequences, the data here suggest that T cell reactivity to the transgenic lines would also be diminished to a similar extent.

With regards to end-use quality, the decreases in mix time in the transgenic lines relative to nontransgenic Butte 86 suggest that the altered protein compositions may have a negative effect on the mixing properties of the flour. However, given the short mix time and tolerance observed in the control, it is difficult determine the significance of the effects. Nonetheless, reductions in SDS sedimentation volumes in the transgenic lines suggest that one effect of the gene silencing was a decrease in gluten strength. This is consistent with the decrease in the ratio of HMW-GS to LMW-GS that was observed in the transgenic lines and is further supported by the observed decreases in water absorption in the transgenic lines. While the most notable difference between the nontransgenic and the transgenic lines was the absence of alpha gliadins, it must be kept in mind that the HMW-GS also showed a small decrease. Because the HMW-GS have a major effect on the functional properties of the flour (Shewry et al., 2003), even small decreases in these proteins could confound the interpretation of the quality data. In other transgenic studies, the effects of the alpha gliadins on flour functional properties were inconclusive. Becker et al. (2012) performed small-scale rheology tests on a mixture of flour from two transgenic lines because of the small amounts of transgenic material that were available and concluded that flour from the transgenic lines did not differ from controls in dough rheology. However, gluten from the transgenic lines had a higher maximum resistance to extension and a lower extensibility than gluten from the control. Barro et al. (2015) examined only SDS sedimentation volumes, but it was not possible to determine whether the increased values observed in their transgenic lines were due to the decrease in alpha gliadins or alterations in the levels of other proteins. In comparison, when omega-5 gliadins or omega-1,2 gliadins were down-regulated in transgenic plants, there was an increase in both mix time and mix tolerance, suggesting that the mixing properties of the flour were improved, and SDS sedimentation volumes were similar or slightly increased (Altenbach et al., 2014b; Altenbach et al., 2019). In the future, it may be interesting to cross transgenic plants in which the alpha gliadins have been eliminated with ones in which the omega-1,2 gliadins have been eliminated and assess both flour quality and IgG and IgA antibody reactivities of the resulting lines.

An important question is whether it will be feasible to target only those alpha gliadin genes encoding proteins with CD epitopes or, alternately, a subset of genes encoding proteins with the greatest numbers of epitopes. To achieve this goal, it will be important first to obtain all of the alpha gliadin gene sequences from the cultivar of interest. The availability of a reference genome sequence from Chinese Spring makes it possible to design gene capture methods to obtain complete sets of gluten protein genes from different cultivars. This might be accomplished using a capture system that includes baits for all high-confidence exons from the International Wheat Genome Sequencing Consortium (IWGSC) genome assembly of Chinese Spring that is commercially available from Arbor Biosciences (Ann Arbor, MI). Alternately, baits for the capture system may be specific for genomic regions encoding the major gluten proteins in Chinese Spring as annotated by Huo et al. (2018a; 2018b) or based on the sequences of all known gluten genes from various Triticeae species and cultivars (Jouanin et al., 2019). However, even with complete sequence information it will be very challenging to identify regions that can be used as triggers in RNAi constructs given the similarities in the sequences of the different genes. Genome editing using CRISPR/Cas9 is an alternate approach that promises greater specificity since it requires only 20 bp of identical sequence for the guide RNAs that determine the sites of the mutations introduced into target genes. In addition, genome editing approaches potentially could be used to alter specific epitope sequences within alpha gliadin genes, as suggested by Ruiz-Carnicer et al. (2019). However, off-target mutations have also been reported in CRISPR/Cas9 edited plants (Endo et al., 2015). Additionally, genome editing is not simple in a family as complex as the alpha gliadins. The method can create indels of various sizes in both expressed genes and pseudogenes that either eliminate proteins or introduce new protein variants. And, when multiple genes are present in tandem in the genome, as in the case of the alpha gliadins, one or more genes may be deleted. Without a doubt, the challenges are many to achieve the long-term goal of reducing the immunogenic potential of wheat. But insight into both the complement of proteins in wheat flour and the roles that different groups of wheat gluten proteins play in determining the functional properties of the flour should make it easier to do so while retaining the unique viscoelastic properties of the flour.

## Data Availability Statement

The mass spectrometry data was uploaded to ProteomeXchange *via* the PRIDE database. The dataset identifier is PXD016930 and 10.6019/PXD016930.

## Author Contributions

SA designed the study, analyzed the data, and wrote the manuscript. AA contributed to study design, assay protocol development, data analysis, and writing of the manuscript. MR was responsible for designing the RNAi construct, transforming the plants, and identifying transgenic lines. H-CC conducted 2-DE analyses. H-CC and AS-B were responsible for MS/MS analyses. XY was responsible for immunoassay experiments and interpretation of data. BS was responsible for end-use quality testing and interpretation of results. PG was responsible for subject recruitment and clinical characterization of patients. All authors contributed to editing of the manuscript and approved the manuscript.

## Funding Sources

This work was supported by the United States Department of Agriculture, Agricultural Research Service CRIS 2030-21430-014-00D (to SBA). Additional support was provided by the National Center for Advancing Translational Sciences, National Institutes of Health, through Grant Number UL1 TR000040 (to AA).

## Conflict of Interest

The authors declare that the research was conducted in the absence of any commercial or financial relationships that could be construed as a potential conflict of interest.

## Abbreviations

AACCI, American Association of Cereal Chemists International; AAI, alpha-amylase inhibitors; CD, celiac disease; ELISA, enzyme-linked immunosorbent assay; ESTs, expressed sequence tags; HMW-GS, high molecular weight glutenin subunits; LMW-GS, low molecular weight glutenin subunits; MS/MS, tandem mass spectrometry; RNAi, RNA interference; 2-DE, two-dimensional gel electrophoresis.
