# Supplementary material for: Reducing the Immunogenic Potential of Wheat Flour: Silencing of Alpha Gliadin Genes in a U.S. Wheat Cultivar
Source: Front Plant Sci. 2020 Feb 25;11:20. doi: 10.3389/fpls.2020.00020 (PMC7052357; doi:10.3389/fpls.2020.00020)
Supplement: Supplementary file 1 [file DataSheet_1.zip › Supplementary File 1.pptx]

## Slide 1
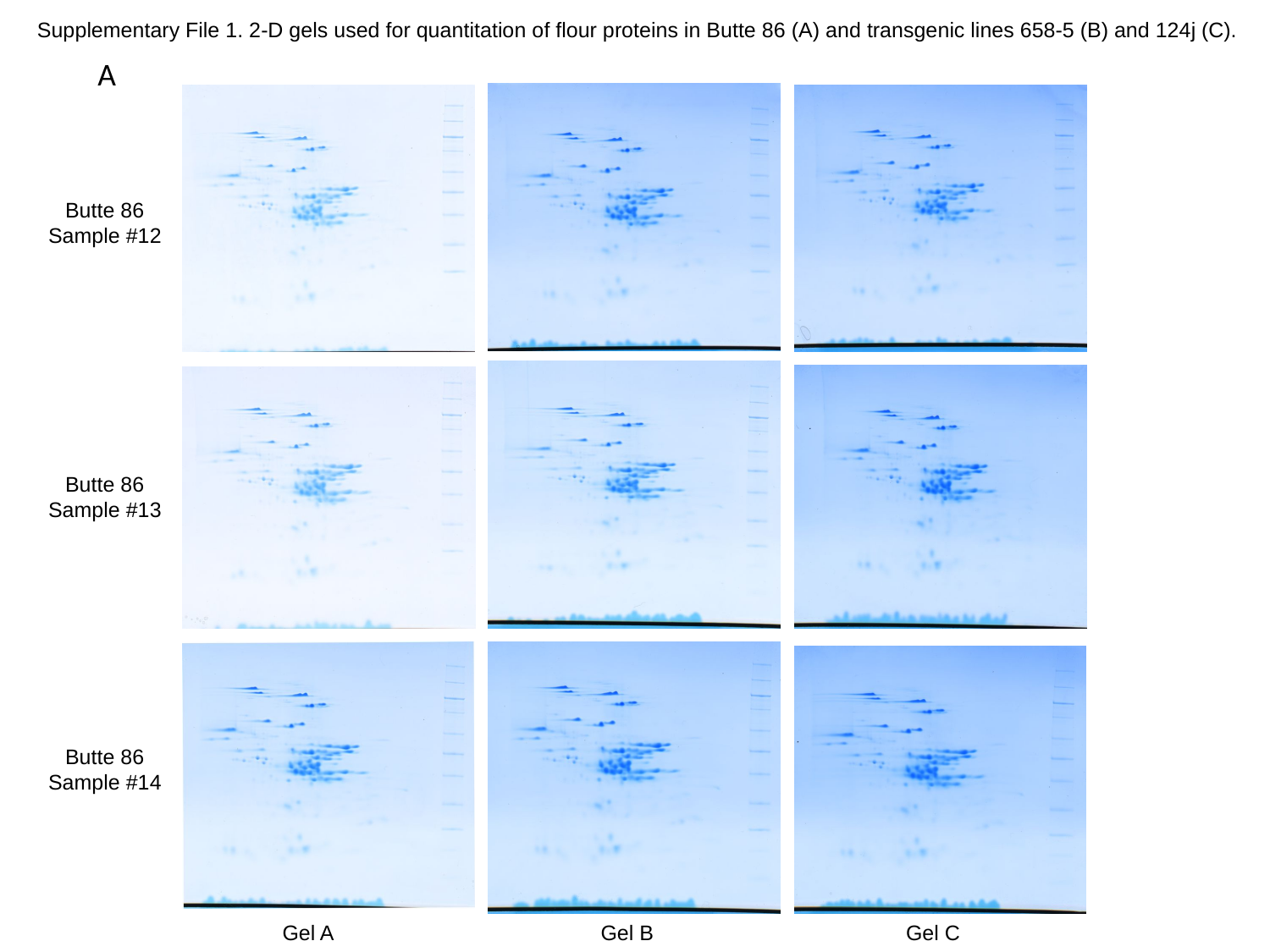

Supplementary File 1. 2-D gels used for quantitation of flour proteins in Butte 86 (A) and transgenic lines 658-5 (B) and 124j (C).
A
Butte 86
Sample #12
Butte 86
Sample #13
Butte 86
Sample #14
Gel A
Gel B
Gel C

## Slide 2
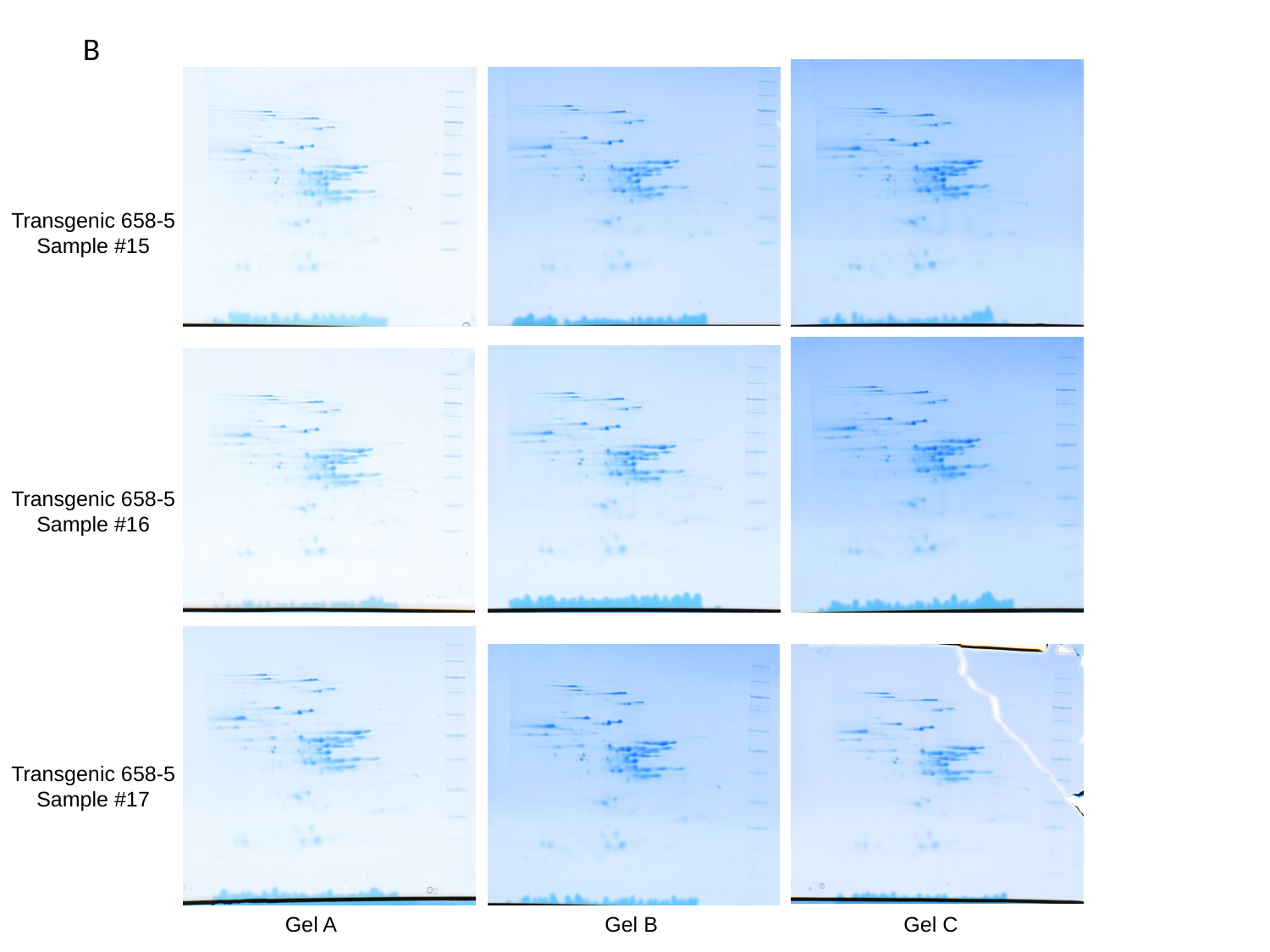

B
Transgenic 658-5
Sample #15
Transgenic 658-5
Sample #16
Transgenic 658-5
Sample #17
Gel A
Gel B
Gel C

## Slide 3
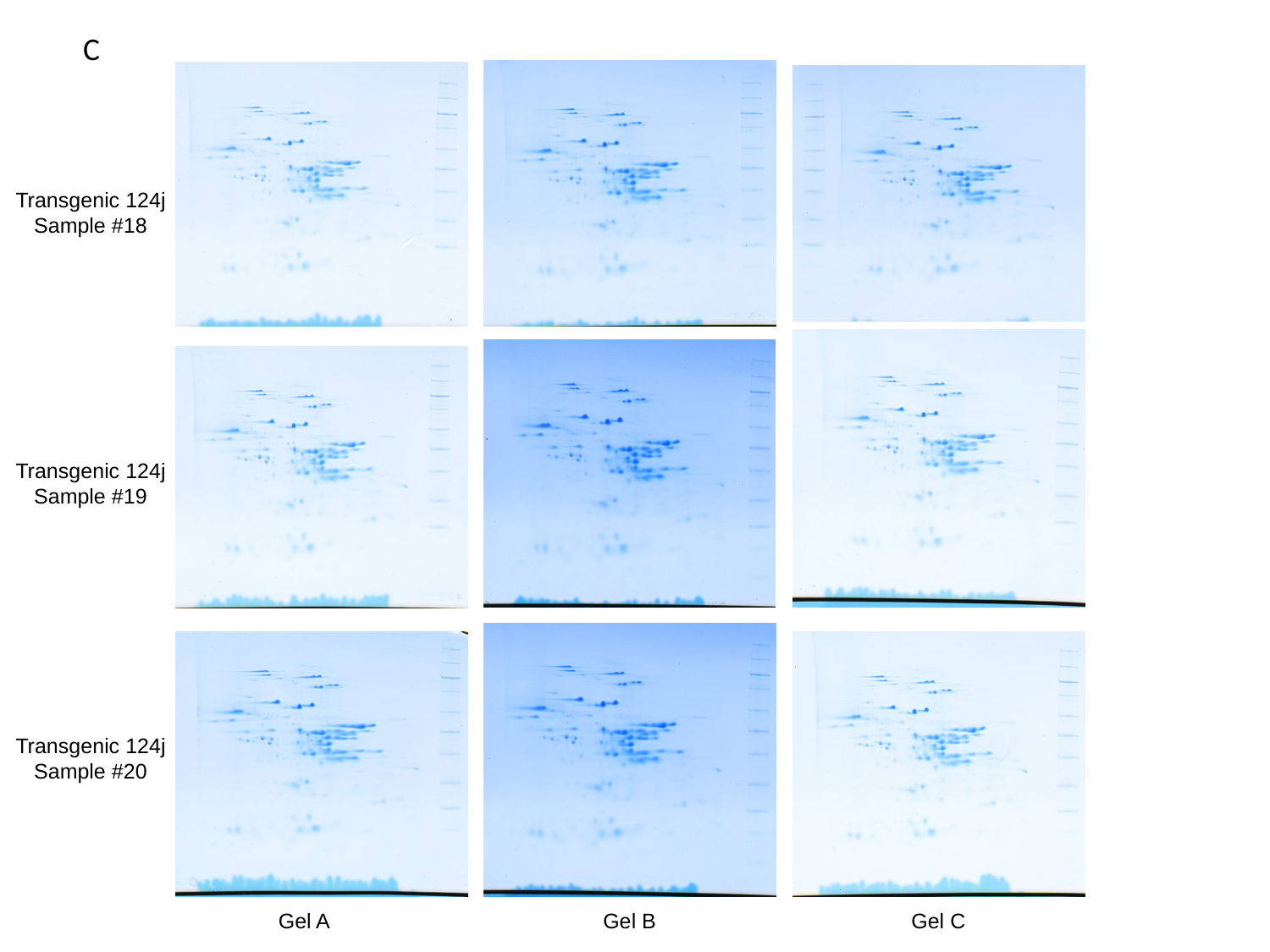

C
Transgenic 124j
Sample #18
Transgenic 124j
Sample #19
Transgenic 124j
Sample #20
Gel A
Gel B
Gel C
